# Why do students drop out of regular sport in late adolescent? The experience of a systematic review

**DOI:** 10.3389/fpubh.2024.1416558

**Published:** 2024-12-16

**Authors:** Yuancai Zhang, Feng Wang, Zsolt Szakál, Zsolt Bíró, Mátyás Kovács, Balázs Őrsi, Karolina Eszter Kovács

**Affiliations:** ^1^College of Physical Education and Health, East China Normal University, Shanghai, China; ^2^School of Physical Education, Fujian Polytechnic Normal University, Fuzhou, Fujian, China; ^3^Kölcsey Ferenc Teacher Training Institute, Debrecen Reformed Theological University, Debrecen, Hungary; ^4^Debrecen Football Academy, Debrecen, Hungary; ^5^Faculty of Chemical Technology and Biotechnology Budapest University of Technology and Economics, Budapest, Hungary; ^6^Institute of Psychology, University of Debrecen, Debrecen, Hungary

**Keywords:** dropout, sport persistence, systematic review, individual factors, social support

## Abstract

**Introduction:**

The positive impact of youth sport on physical, mental and social health has been highlighted in several research which reinforces further investigations concerning the reasons for dropout of athletes. As one of the most emergent difficulties in youth sports is to prevent athletes from dropping out, it is important to explore what factors play important part in this process. The purpose of this study was to identify barriers and challenges related to sport persistence and dropout.

**Methods:**

We conducted a systematic literature review using the EBSCO Discovery Service Search Engine. Our method followed the Preferred Reporting Items for Systematic Reviews and Meta-Analyses (PRISMA) guidelines.

**Results:**

Out of the initially examined 512 publications, 11 papers were included in our final sample. Original empirical research published in a peer-reviewed journal, papers focusing on participants age below 25 years and exploring factors influencing dropout determined by any levels of Bronfenbrenner’s model were analyzed. Results showed that gender differences were extensively examined and did, in fact, contribute to dropout ratios, while socio-cultural and ethnic backgrounds were largely disregarded in most studies. From presented individual psychological factors perception of ability and success, enjoyment, sports satisfaction and life satisfaction all protected against dropout.

**Discussion:**

Achievement, goal, social and win orientation emerged as positive predictors of sport persistence in many studies, along with motivation and commitment. Many non-psychological factors were also mentioned. Sports requiring more training may lead to increased dropout rates, just as well as the lack of knowledge acquisition and competitiveness. Lastly, social environment and status also often play a significant role in dropout. Individuals with more resourceful socio-economical background find it easier to keep up the pace and have the opportunity to participate in more wealth-consuming sports. Social support received from the family and peers is a very strong preventive factor against dropout and both the personality and leadership of the coach were mentioned in multiple studies. A lack of methodological diversity, paired with the presence of only cross-sectional studies fulfilling the inclusivity criteria raises attention to the importance of examining underrepresented factors and the need for longitudinal research on the topic.

**Systematic review registration:**

https://inplasy.com/inplasy-2024-11-0015/, INPLASY2024110015.

## 1 Introduction

Continuous and persistent participation in sport has a pivotal role in supporting the physical, mental and social health of the individuals (1–4). Therefore, focusing on the topic of sport persistence and dropout has multifaceted benefits regarding public health (5–7). However, although the prevalence of regular physical activity is relatively high in childhood, further participation in sporting activities decreases with age (8, 9). Therefore, nowadays, one of the key issues in youth sports is what methods and tools can be used to keep the athlete in his chosen sport in the long term and how to prevent or reduce the chance of early dropout from the sport. The causes of this phenomenon can be, among others, the quality of the athlete’s relationship with the coach or parents (10) and the quality of the motivational atmosphere created by the sports socialization environment (11). This paper seeks to examine previous research focusing on dropout from sports. Our investigation intends to delve into the various factors that affect dropout among student-athletes, applying Bronfenbrenner’s ecological model, which encompasses intrapersonal, interpersonal, and environmental influences. A core premis of Bronfenbrenner’s model is the articulation of human beings and their environment and the interplay such have. In more detail, understanding behavior of the individual is a four dimensional process based on micro-, meso-, exo-, and macro systems according to Bronfenbrenner. In the concentric rings of the model, the central circle is referred to as the self. The microsystem is inclusive of a web of close people defining the participant’s world including family, friends, work and school environments. The mesosytem is the second layer and illustrates situations, where the components of the microsystem interact and surroundings that connect those different microsystems. The exosocial system, which is the third layer, deals with peripheral relation where the individuals are more of observers than participants. In relation to sports participation, exosystem elements include the institutional and physical features of the sporting context like the community, parks, recreation and sports centers (6, 7, 12). The macrosystem represents the fourth and largest layer in Bronfenbrenner’s model which encapsulates an overall uniformity of society pertaining to the previously established systems (micro, meso and exo).

Dropout has two meanings. According to the first interpretation, dropout in elite sports means that the sports career ends before the athlete can reach the peak of their performance. This type of dropout is a typical phenomenon among child and adolescent athletes (13, 14). In contrast, according to the other interpretation, ending a career after reaching peak performance is called retirement. In general, retired athletes are older than those who dropped out from the same sport, according to this first interpretation (15, 16). If we examine dropouts from recreational sports, in this case, the concept means that the individual stops exercising for their own entertainment or to satisfy their needs, for example, in a club or fitness center. In health-related sports, dropout means that an individual leaves an exercise or rehabilitation program for any reason before the end of the program (17, 18). In this sense, dropout can occur at any age and is characteristic of the premature termination of any supervised physical activity (19). Unlike elite sports, the latter two organizational levels participants did not exercise for a sports career or to achieve their peak performance (20). In the present case, we would like to examine the emphasis by focusing on the first report.

Several studies have been published on the international scene that provide valuable information about the reasons for participation in sports and their importance, but much less deal with the investigation of the reasons for youth dropout from sports (21–25). According to Gould and Horn, 33% of youth between the ages of 10 and 17 drop out of organized sports yearly. This affects many millions of young people worldwide (26). Many factors can cause young people to quit sports, including incompatibility with teammates, lack of success or expected development in the sport, confrontation with the coach, lack of fun, and other reasons (27). Getting to know the causes of dropout in sufficient depth is necessary so that sports professionals working in youth sports can design sports programs and sports experiences for the participants that meet the needs of the athletes and contribute positively to their personal development (28).

Dropout can be regarded a complex phenomenon with multiple causes (29, 30). It can be examined from the point of view of the sports system and the athlete as well. From the point of view of the sports system, dropout cannot be seen as something that could have been avoided if only the athlete had been more motivated and the environment had supported them better (31). Therefore, attrition is considered a loss of talent and a lost investment from an economic point of view (32). From the athlete’s point of view, attrition can be a source of negative feelings that can lead to a transition to a career outside of elite sport (15). Therefore, both research and applied sports psychology deal with the causes of dropout, its prevention, and the efforts made by athletes in this field. Dropout may happen due to a deliberate decision by the athlete, for example, after not seeing a future in the sport due to a lack of performance improvement. On the other hand, it can also come from a forced decision due to a career-ending injury. Research shows that it makes a significant difference to an athlete whether the decision to drop out is voluntary or involuntary. It is very common for athletes to manage the transition to post-career more easily if they feel that retirement from sport was a voluntary decision (10). The difficulty of coordinating school studies and sports are also often mentioned as a main reason behind dropout. In many cases, students feel that they can no longer combine their schoolwork with the high demands of sports training and competitions. Therefore, they end their sports careers in order to prioritize their studies (19, 33). Young athletes may also realize they have no chance of reaching the top and see further career investment as a waste of time (34, 35). This feeling can be exacerbated by declines in performance - especially during or after puberty - and motivational crises, especially after injuries. In addition, if the athlete even feels that he is not receiving adequate support and attention from the coach, nothing will stop them from leaving the training group for all the reasons mentioned above (36).

The study by Witt (36) classifies the reasons for dropping out of sports into three groups. Their study blames intrapersonal reasons first, interpersonal reasons second, and structural reasons third. Based on their research results, we can conclude that, on an intrapersonal level, the enjoyment of sport ceases due to excessive expectations often set by the performance-oriented system. Then, the lack of results and thus the continuous goals of failure, and later, these factors can cause them to give up their previous lifestyle (37). On the second interpersonal level mentioned in the research, there probably needs to be sufficient support in the parental and educational environment (38, 39). At this age, of course, the peer group also plays an important role, making it increasingly apparent how many sacrifices regular sports involve. Young people can increasingly feel that while their peers can spend their free time having fun, they have an obligation to go to training, which can create a sense of compulsion in the athlete (40–42). The structural level mentioned as the third cannot be neglected, according to which athletes can at any time suffer an injury to such an extent that they must immediately stop their previous activities (43, 44). Of course, the latter rarely occurs, but minor sports injuries or possibly more frequent injuries can hinder the athlete’s preparation, causing their results to fall short. In the case of team sports, they may be excluded from the starting lineup (45).

In this systematic review, our objective is to scrutinize past studies about dropout from sport. Our research aims to explore factors influencing dropout from sport among student-athletes, utilizing Bronfenbrenner’s ecological model, which includes intrapersonal, interpersonal, and environmental factors. This model offers a more nuanced understanding of the concept of sport persistence. To achieve our aim, we have formulated the following research question: What sort of factors can be identified to have a significant effect on the dropout from sport regarding the individual, micro-, meso-, and macro system levels among athletes younger than 25 years?

## 2 Methods

In our study, we aimed to conduct a narrative synthesis for several reasons. First, we hypothesized that we would experience a significant heterogeneity in the included studies. The diverse methodological background applied in the papers makes it challenging to assess the results quantitatively. Also, we also believed that the number of studies will be low. In cases with few studies, especially if each has a small sample size or limited statistical power, a meta-analysis may lack robustness while a narrative synthesis can offer a clearer understanding by focusing on themes or trends without requiring statistical aggregation. Lastly, our aim of identifying research gaps also supported using the methodology of narrative synthesis (46). The systematic literature review follows the Preferred Reporting Items for Systematic Reviews and Meta-Analyses (PRISMA) guidelines (47, 48). The review is registered in the International Platform of Registered Systematic Review and Meta-analysis Protocols (INPLASY)^1^^,^^2^.

### 2.1 Literature search

Systematically searches were conducted in EBSCO Discovery Service Search Engine, which contains 85 databases. The keywords we used for searching were (“sport dropout” [Abstract] OR “dropout from sport” [Abstract] OR “discontinuation of sport” [Abstract]) AND (“influential factors” [Abstract] OR “individual factors” [Abstract] OR “intrapersonal factors” [Abstract] OR “Interpersonal factors” [Abstract] OR “environmental factors” [Abstract] OR “institutional climate” [Abstract]). Searches were conducted in English on 16 December 2023, resulting in a total of 512 records. After a double filtering process, 51 of these records were excluded, leaving 461 records. Abstract and title screening removed an additional 389 records, leading to 72 papers that underwent full-text screening. Ultimately, 11 papers were included in the qualitative synthesis.

### 2.2 Inclusion and exclusion criteria

The following inclusion criteria were set, following the PICOS format (P, Population; I, Interventions; C, Comparisons; O, Outcomes):

- Population: age below 25 years (due to focusing on children and adolescents, more specifically students learning in primary, secondary or tertiary education) and participating in any kind of sport (competitive or recreational).
- Intervention: original empirical research published in a peer-reviewed journal.
- Comparison: manifestation of dropout from sport and factors influencing it determined by any levels of Bronfenbrenner’s model.
- Outcomes: any kind of intrapersonal, interpersonal and environmental factors contributing to the dropout from sporting activity of the child and/or adolescent.
- Written in English language.
- In disciplines of education, psychology, health, social sciences and humanities and sports sciences.

In this study, reviews, commentaries, letters to the editor, conference papers, books, book chapters, dissertations and newspaper articles were excluded. Also, papers focusing on non-healthy participants were not used for analysis.

### 2.3 Data extraction and assessment of methodological quality

We performed a multistage screening process to select studies which met the inclusion criteria. After removing the duplicate studies, a multistage screening process was applied to select those studies which met the inclusion criteria:

- Stage 1, title and abstract screening: the first review author screened the titles and abstracts of all identified records (KEK). Twenty-five per cent of all titles and abstracts were independently assessed by a second review author (ZsSz, ZsB, MK, BŐ). At this stage, all studies whose adequacy was questionable were taken forward to the full-text screening at this stage.
- Stage 2, full text screening: two review authors (ZsSz, ZsB, MK, BŐ) independently screened all full texts. In cases of uncertainty, the other authors also checked the decision.

The establishment of clear inclusion and exclusion criteria before beginning the review helped the authors to achieve consensus on questionable papers. We resolved disagreements through discussions, each reviewer presenting their perspective, and they deliberate until reaching an understanding. For data extraction, an Excel spreadsheet and Data Extraction Forms were applied. We included full article citation, study objectives, study design, how the study attempted to avoid bias, participant characteristics and numbers, type of sport, level of sport, results/outcome and comments related to study quality.

### 2.4 Risk of bias

The quality of the studies was evaluated by the Joanna Briggs Institute (JBI) critical appraisal tool (49). This tool assesses various aspects of study design, conduct, and reporting to gage the reliability and validity of findings. It considers factors such as randomization, blinding, sample selection, and data analysis methods. By identifying potential sources of bias, researchers can better interpret study outcomes and make informed decisions about the applicability of evidence in healthcare practice. This measure aids in promoting transparency, rigor, and credibility in research, thereby enhancing the quality of evidence-based healthcare interventions and guidelines. Papers were evaluated according to the appropriate tool on a 4-point scale (yes/no/unclear/not applicable) (Table A1).

Regarding the inclusion criteria (Q1), most studies provided clearly defined inclusion criteria, which reduces the risk of selection bias by ensuring consistency in participant selection. The majority of studies detailed their subjects and settings Q2, improving replicability and external validity. Across studies, exposure measurements (Q3) were generally considered valid and reliable, enhancing internal validity. This criterion ensures that studies accurately capture relevant data on factors like sport dropout. Most studies utilized objective, standardized criteria (Q4) for measuring dropout-related conditions, with few exceptions. This consistency strengthens the comparability of outcomes across studies. The criteria of the identification of confounding factors (Q5) was met by only a subset of the studies, which identified potential confounders that could affect dropout rates, such as socioeconomic background or level of coaching support. Based on this, several studies failed to provide strategies for handling confounding variables (Q6). However, outcome measurements were generally valid and reliable across studies (Q7), ensuring accuracy in identifying dropout factors. Consistent, reliable outcome measurement reduces measurement bias and supports more robust conclusions. The studies largely utilized appropriate statistical analyses for their data (Q8), which supports accurate interpretation of results. Overall, most studies could be categorized as low-risk studies, exhibiting strong methodological rigor, enhancing confidence in their findings regarding factors influencing sport dropout. Some studies were assessed as moderate-risk studies due to missing criteria, particularly around confounder management. This could mean that certain biases may have influenced their outcomes.

## 3 Results

**Figure 1:**
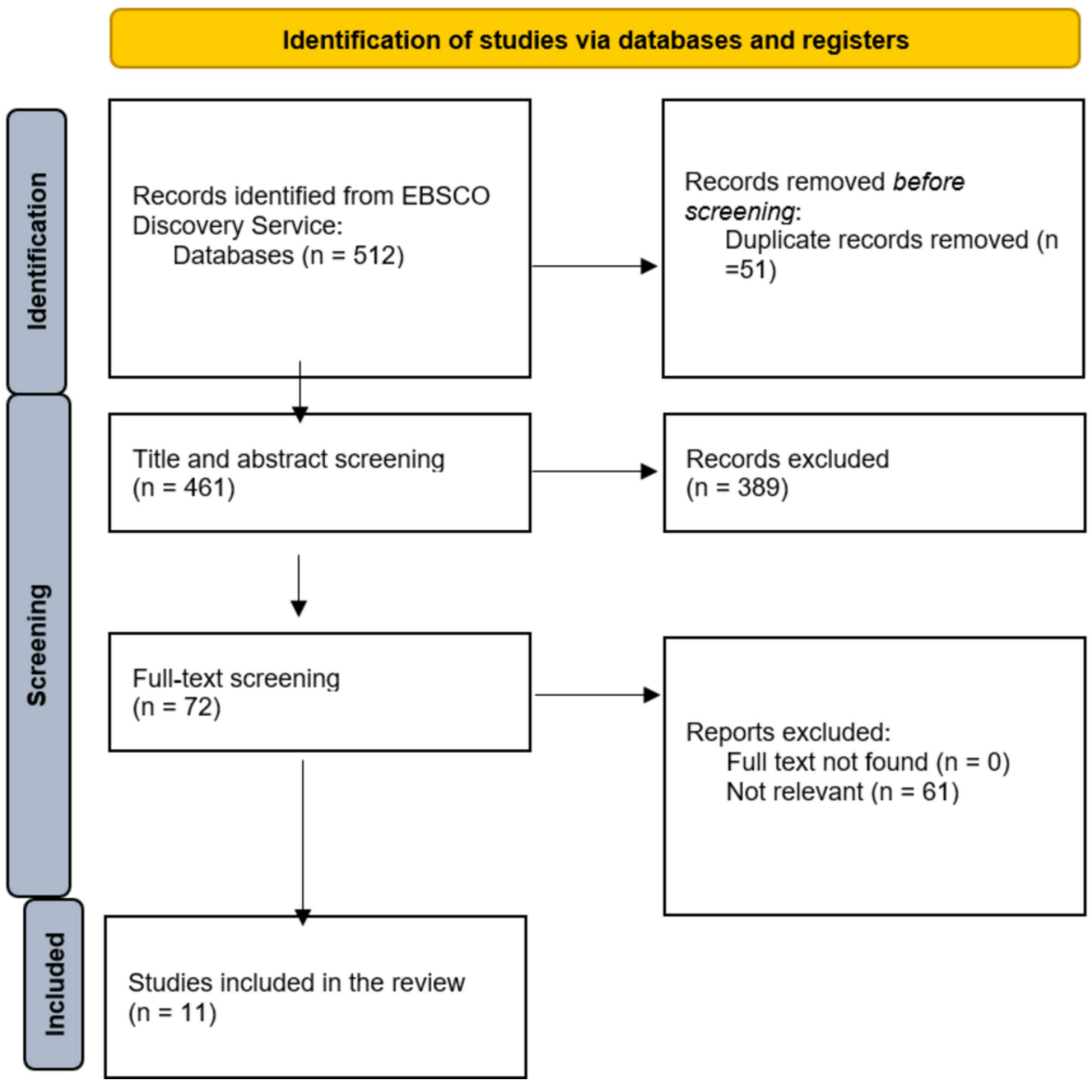
Preferred reporting items for systematic reviews and meta-analyses (PRISMA) diagram.

Overall, the systematic search yielded a total of 512 records. Firstly, title and abstract screening was carried out, which led to keeping 62 records for full-text screening (Figure 1). All of these were examined for eligibility. A total of 11 articles met the criteria (Table 1). The articles were published between 1988 and 2021, approximately 1/3 of the papers were published after 2010 (50–53). Regarding the territorial distribution, most studies focus on one particular nation or country and only one paper focuses on two countries (52). Spain was the most frequently appeared country since three investigations were carried out in this country (51, 52, 54) along with France (55–57). Two studies were carried out concerning the United States (58, 59). One study was introduced in regard of Canada (60) and Germany (50), Slovenia (53) and Italy (52). Therefore, research carried out in Europe is overrepresented. Also, there is a research gap regarding the international comparison of dropout. Regarding the research methods, quantitative studies were significantly more popular concerning the type of the investigation since 10 out of 11 papers introduce the results of quantitative studies and only one paper reflects on the results of interviews as a qualitative study method.

**Table 1:** Papers involved in the analysis.

	Country	Age group	Mean age	Level	Type of sport	Research method	Number of participants
Baron-Thiene & Alferman (50)	Germany	Adolescent	*M* = 16.2 (SD = 0.65)	Elite athlete	General (track and field, swimming, and diving in the summer and cross-country skiing, biathlon, and ice skating + basketball, handball, soccer, and volleyball)	Quantitative - questionnaire	125
Calvo & Topa (51)	Spain	Adolescent	*M* = 14.3 (SD = 1.6)	Elite athlete	Football	Quantitative - questionnaire	492
Cervelló et al. (54)	Spain	Adolescent	*M* = 15.23 (SD/N/A) between 14 and 18 years	Elite athlete	General (mainly tennis)	Quantitative - questionnaire	134
Consoni et al. (52)	Italy and Spain	Adolescent	N/A (14–18 years)	Non-elite athlete	General	Quantitative - questionnaire	614
Duda (58)	USA	Young adult	*M* = 20.97 (SD=N/A)	Non-elite athlete	Basketball, volleyball	Qualitative - interview	134
Fraser-Thomas et al. (60)	Canada	Adolescent	*M* = 16.4 (SD = 2.6) + M = 18.3 (SD = 4.1)	Elite athlete	Swimming	Quantitative - questionnaire	20
Guillet et al. (55)	France	Adolescent	*M* = 17.06 (SD = 1.32) + M = 15 (SD = 0.81)	Elite athlete	Handball	Quantitative - questionnaire	723
Le Bars et al. (56)	France	Adolescent	*M* = 17.9 (SD = 1.3) + M = 16.7 (SD = 1.1)	Elite athlete	Judo	Quantitative - questionnaire	186
Lea & Branco (53)	Slovenia	Adolescent	*M* = 14.87 (SD=N/A)	Elite athlete	Athletics (javelin, high jumper, runner, sprinter)	Quantitative - questionnaire	566
Ryan et al. (59)	USA	Young adult	*M* = 21 (SD=N/A)	Non-elite athlete	Aerobic, Tae Kwon Do	Qualitative - interview	40
Sarrazin et al. (57)	France	Adolescent	*M* = 14.07 (SD = 0.79)	Elite athlete	Handball	Quantitative - questionnaire	335

### 3.1 Individual non-psychological variables in the background of dropout

Firstly, we investigated the individual non-psychological variables mentioned in the papers. We started the exploration of the sociodemographic variables. *Gender differences* were detected in exhaustion and somatic complaints which was higher among women. Consoni et al. (52) reported that a larger drop-out emerged in females than in males. According to Duda (58), males who had competed in sport emphasized social comparison-based goals significantly more than females who had competed in sport. Not relevant impact was shown by the research of Le Bars et al. (56). Guillet et al. (55) and Sarrazin et al. (57) investigated only female handballers where no gender impact could be seen. In the other cases (51, 53, 54, 59, 60), the role of gender was not measured.

The outcomes regarding *socio-cultural and ethnic backgrounds* were hardly integrated into the studies. In the research, the social status of the participants or their offspring was not explicitly specified. Only Consoni et al. (52) acknowledged that they studied adolescents who were mostly from the middle class, however, they did not measure the effect of social status. As for the subject of dropout from sport, there is a lack of research on the importance of social status and ethnicity, so we can only rely on information about its effect on athletic motivation and habits.

Concerning sport-related demographical variables, we could detect the overrepresentation of factors reflecting on *elite sport*. Sport biography (50, 58), year of experience and career length (53, 58) also refers to carrier-related factors. Also related to competitive sport, placing (53) and level of competition (50) are mentioned to be significant impact on sport achievement and the likelihood of dropout. Exercise participation level (50) as well as number of sporting hours (50, 53) and training patters (60) can be categorized as individual non-psychological variables reflecting on type of behavioral commitment (Table 2).

**Table 2:** The factors influencing dropout from sport in the various levels of the ecological model.

The role of the individual, micro-, meso-, and macro - system in dropout		
Individual level	Micro level	Meso level
Non-psychological factors Gender (52, 55, 56, 58, 60) Socio-cultural and ethnic background (52) Sport-related demographical variables: sport biography (50, 58), year of experience / career length (53, 58), placing and level of competition (50, 53), exercise participation level (50), number of sporting hours (50, 53), training patters (60)	Coach-related factors Leadership style (autonomy-supportive as protective factor) (55, 60) Motivational climate (task-involving as protective factor) (54, 55, 57)	Climate-related factors Ego- and task-oriented organizational climate (54–56) Coach’s mastery and competitive climate (55)
Psychological factors Personality: perception of ability (50, 54, 55), physical self-perception profile (56), perception of success (54, 56), enjoyment (55, 59), satisfaction with sport practice (51), life satisfaction (50), openness (50), exhaustion (50) and energy deficit (50) Motivation: extrinsic and intrinsic motivation and amotivation (51, 57–59), Self-determination theory appeared relatedness: (55, 57); competence: (54–57, 59); autonomy: (55, 57), body-related motivation (59), commitment [personal investment, social constraints, involvement opportunities, valuable opportunities and other priorities] (55). Orientation: achievement goal orientation [athlete’s task-orientation and ego-orientation] (50), social orientation (50), win-orientation (50) and volition (50), future sport intentions (57) Learning and development: learning strategies [cognitive-metacognitive and affective motivational] (52), development/progress (55), positive feedback (55), the athlete’s pursuit of learning (56), the athlete’s pursuit of comparison (56–58) Health: Physical health complaints (50), worry about health (50)	Peer-related factors Teammates to facilitate personal investment (55, 60) Task-oriented and ego-oriented perception of peers (54, 57) Family-related factors Parents’ investment (60) Parental support (60) Sibling influence (60)	

### 3.2 Individual psychological factors affecting dropout

After the categorization of the individual non-psychological factors, we continued the detection of the psychological variables. As a result of the investigation, five groups of variables were detected, including personality, motivation and commitment, orientation, learning and development and health.

*Personality* is significant concerning sport-related behavior and sport achievement as well. Therefore, it is not surprising that most research emphasized dropout as an outcome of a personality-related variable. In this regard, perception of ability (50, 54, 55) and physical self-perception profile (56) were emphasized as protective factors against dropout. Perception of success was also referred as a factor having a significant negative impact on dropout (54, 56). The evaluation of feelings also appeared in this context. As positive emotion-related variables, enjoyment (55, 59), satisfaction with sport practice (51), life satisfaction (50) and openness (50) could be seen, anticipating persistence and protecting from dropout. On the contrary, the effect of the negative feelings such exhaustion (50) and energy deficit (50) are risk factors regarding dropout.

As the second biggest group, *motivation and commitment* was detected. Motivation included extrinsic and intrinsic motivation and amotivation (51, 57–59). Related to motivation, the Self-determination theory appeared in more studies relatedness: (55, 57); competence: (54–57, 59); autonomy: (55, 57). As specific outcome, body-related motivation (59) was explored as a factor having a significant impact on persistence. Commitment is also proved to be a protective factor regarding dropout (55). It included personal investment, social constraints, involvement opportunities, valuable opportunities and other priorities (55).

Besides motivation, various types of *orientation* appeared in the papers. As supposed, mostly achievement goal orientation [athlete’s task-orientation and ego-orientation] (50) could have been detected as the most important way of orientation. Besides, social orientation (50), win-orientation (50) and volition (50) seemed to be significant and protective factor against dropout. Future sport intentions (57) also appeared, highlighting the positive impact of planning and positive future-related thoughts.

*Learning and development* are also relevant concerning long-lasting sporting activity, including learning strategies [especially cognitive-metacognitive and affective motivational] (52), development/progress (55), positive feedback (55), the athlete’s pursuit of learning (56), and the athlete’s pursuit of comparison (56–58). All of these variables can be regarded protective factors against dropout.

Last but not at least, the relevance of *health* was detectable. Physical health complaints (50) and worry about health (50) could have been seen as variables influencing dropout, more precisely anticipating dropout from sporting activity. Therefore, we could see that worse health and injuries leads to higher likelihood of dropout.

### 3.3 The role of the micro-system in dropout

Concerning the micro-system level factors connecting to dropout, coach, family and peer-related factors could have been categorized. Unsurprisingly, *coach-related factors* play a significant role in athletic performance. The leadership style and interpersonal behavior of the coach are particularly important. An autonomy-supportive leadership style, regardless of whether it is exhibited by teachers, parents, coaches, school administrators, or healthcare professionals, can foster self-determined regulation and persistence. Conversely, a controlling style can undermine self-determination (55, 60). According to Sarrazin et al. (57), a task-involving motivational climate leads to a greater sense of competence, autonomy, and relatedness, while an ego-involving motivational climate does not result in positive impacts. This finding is also evident in studies that examine the task-oriented and ego-oriented perception of coaches (54, 57). Lastly, besides the athlete’s personal investment, the coach’s investment is of paramount importance as well, decreasing the likelihood for dropout (55).

The study’s focus on adolescents and young adults necessitates an examination of the role played by *peers and teammates*. The influence of peers in sports is often linked to increased levels of motivation, commitment, and persistence and its lack can increase the chance of dropout (60). Coaches and athletes alike recognize the importance of teammates’ perceived investment in the sport (55), as it can encourage greater personal investment from the athlete. Peer attitudes were also found to be relevant to the study, with task-oriented and ego-oriented perspectives observed in several papers (54, 57). These attitudes are closely linked to the overall climate of the team.

It is unsurprising to note that *family-related factors* could be classified as a third major group. This is due to the fact that family serves as the primary area of socialization during childhood and continues to hold significant influence during adolescence and early adulthood. Therefore, parents’ investment (55) and parental support (60) have a significant decreasing impact on dropout from sport. Besides, the role of the siblings can also be huge as sibling influence can also support commitment (60).

Climate can be regarded as a meso-level variable. It encompasses not only the athlete’s immediate surroundings [known as micro-level variables] but also the interplay between these environmental factors. Research papers have highlighted two types of climate from the athlete’s perspective: ego-oriented and task-oriented climate (54–56). The former emphasizes social comparison and striving to demonstrate superiority over others, while the latter prioritizes individual and collective improvement, learning, and mastery of skills. An optimal balance of both types of *climate* has been shown to enhance the athlete’s level of sport persistence and the decrease of the likelihood for dropout. On the coach’s side, a coach’s mastery climate (55) can also be detected. This type of climate is characterized by a focus on skill mastery and predicts psychological need satisfaction, self-determined motivation, and commitment to sport.

## 4 Discussion

Investigating dropout from sports is crucial for fostering a culture of sustained sports participation, promoting health and well-being, and maximizing the benefits that sports offer to individuals and society as a whole. It helps create more inclusive, accessible, and supportive sporting environments, ultimately leading to a healthier and more active population.

In certain circumstances, particular sports may experience higher attrition rates among one gender due to a variety of cultural and societal influences. For instance, girls may encounter distinct barriers when participating in certain sports in contrast to boys. Gender-based attrition in sports can be influenced by a variety of cultural, societal, and systemic factors. Traditional gender norms can shape perceptions of what is considered appropriate or expected behavior for individuals based on their gender. These norms may discourage or encourage participation in certain sports (61). If there are few visible and successful role models of a particular gender in a sport, it can lead to reduced interest or belief in one’s own potential to excel in that activity (62). Besides, some sports may have historically been less accessible to one gender due to factors like limited facilities, resources, or opportunities in certain regions or communities, and also, in some cultures, certain sports may be associated with specific gender stereotypes, which can deter individuals from participating if they feel it goes against societal expectations (63). Family and peer attitudes toward certain sports can play a significant role in an individual’s decision to continue or discontinue their participation (64) and on the other hand, policies and practices of sports organizations can either encourage or discourage participation from a particular gender (61). Understanding these cultural and societal influences is crucial for addressing gender-based attrition in sports. Efforts to promote inclusivity, challenge stereotypes, provide equal opportunities, and create supportive environments are all important steps toward ensuring that individuals of all genders have the chance to participate and thrive in sports. Therefore, although this systematic review did not find papers directly focusing on the impact of macro-level factors on dropout, we should emphasize that cultural and societal attitudes toward certain sports can influence dropout rates as mentioned above.

Regarding individual non-psychological variables, the type of sport should be mentioned first that can have a significant impact on dropout. Some sports are more physically demanding than others. Sports that require extensive training, high levels of endurance, or specialized skills may lead to higher dropout rates (65). Athletes may find it challenging to keep up with the demands, leading to burnout or injury. High-level training often requires substantial time investments, which can be difficult to sustain alongside other responsibilities like work, education, or family commitments. Intensive sports often involve repetitive and physically demanding movements, which can lead to overuse injuries or other physical strains, potentially causing athletes to reconsider their participation (66). Also, sports that can be enjoyed over a lifetime [like swimming, cycling, or running] may have lower dropout rates compared to sports with a limited competitive window [like gymnastics or figure skating] (66). Related to this phenomenon, research highlighted that personal factors such as perfectionism and a singular focus on athletic involvement are precursors to burnout (67, 68). The intense competitive nature of these sports can lead to psychological stress, burnout, and mental fatigue, particularly if athletes feel constantly pressured to perform at their peak. Otherwise, in some cases, the environmentally constrained training facilities [snow, mountains, and rural areas] may make the relationship between sport and other activities [e.g. learning] more difficult (50). Athletes may reach a point where they need to transition to other phases of life, such as pursuing education, starting a career, or focusing on family, which can make it challenging to sustain the demands of high-level sports.

During adolescence and early adulthood, the acquisition of knowledge is crucial. Insufficient development of affective skills [such as anxiety and emotional regulation], motivational skills [such as self-perception of competence and causal attribution], and volitional skills [such as perseverance] can directly lead to discontinuing participation in sports. This decision has a significant impact on academic performance since sports and academic achievements are interdependent. Students who quit sports miss out on the positive effects that sports have on academic skills essential to the learning process, which are widely studied in academic achievement research (69). Moreover, participation in sports can also enhance these skills, while a lack of them may lead to early withdrawal from sports (70).

Regarding intrapersonal factors, the relevance of positive psychological variables must be mentioned as preventive factors in dropout from sport. Baron-Thiene et al. (50) stated that career persistence is supported and enhanced by self-optimisation skills of athletes. Also, the level of competitiveness in a sport can be a factor. Athletes who feel overwhelmed by the pressure to perform at a high level may be more inclined to quit (71). Other priorities must be also mentioned. It is important to note that different results can also be assumed for academic performance at different levels of sport participation. Participation in sport is not an exclusionary factor for achieving good academic performance. Some research has shown that elite athletes also excel academically, but this is mainly the case in higher education (72). However, in many cases, elite sport is not the case for academic excellence. This may of course be due to a number of factors. The primary task of the athlete is excellence on the playing field, be it individual or team. In their case, the requirement is not daily physical education, but a daily program of hard training. In addition, it is very difficult for the athlete to devote time or energy to learning, and even if this is partially achieved, the energy invested does not necessarily translate into grades or academic performance.

On the border of intra- and interpersonal variables, resources are also important. Certain sports require specific facilities, equipment, or coaching expertise that may not be readily available to everyone. If a sport is expensive or not accessible in a particular area, individuals interested in that sport may be more likely to drop out. This may be in relation with the social status of the athlete or his/her family. Participation in sports often comes with costs, including equipment, training fees, travel expenses, and competition fees. Athletes from lower socio-economic backgrounds may face challenges in covering these costs, which can lead to dropout (32). Affluent families may have more resources to invest in high-quality coaching, facilities, and training programs (9). This can provide their athletes with a competitive advantage and increase the likelihood of success and continued participation. Families with limited resources may prioritize education over sports, especially if they see it as a pathway to future opportunities (73). This can lead to athletes dropping out to focus on their studies. Affluent families may have more extensive networks that can provide mentorship, sponsorship, and other forms of support that can be instrumental in an athlete’s success and longevity in a sport (74).

Team sports may have unique social dynamics. If an athlete does not feel comfortable or connected with their team members, they may be more likely to drop out. The supportive role of the peers was presented in several papers (75–77). In individual sports, personal motivation and discipline become crucial which were detailed in research focusing on sport persistence. Research has consistently highlighted these factors as key determinants of long-term success and participation in individual sports. Athletes must have a genuine passion and desire to excel in their chosen discipline. This internal drive can help them overcome challenges, setbacks, and moments of self-doubt. Setting specific, measurable, achievable, relevant, and time-bound (SMART) goals is crucial in individual sports. It provides athletes with clear objectives to work toward, which can enhance their sense of purpose and direction (78). Therefore, athletes are more likely to stick with a sport if they find it enjoyable and see benefits, whether those are physical fitness, social interaction, or personal achievement (79, 80).

Their role of the coach and their leadership style was also presented. Young people’s experiences, both positive and negative, with a coach’s leadership style and the team’s motivational climate can have a determining influence on their lives. Coaches often serve as role models for young athletes. Their leadership style, behavior, and values can shape the way young individuals perceive authority figures and influence their own behaviors and attitudes (81). Regarding the leadership style, the autocratic approach is deemed unfavorable as it results in lower satisfaction among players (82). This indicates that our teams prioritize an environment that encourages participation, cooperation, and communication among all members. A coach’s leadership style and the team’s motivational climate can directly impact a player’s skill development. Positive coaching techniques can enhance learning and skill acquisition, while negative experiences can hinder progress (82, 83). Positive coaching experiences can boost a young person’s confidence in their abilities, both in sports and in other areas of life. On the contrary, negative experiences can erode self-confidence (84, 85).

Regarding methodological concerns, it is important to note that only cross-sectional studies were identified, with the exception of one interview study. No randomized controlled trials [RCTs], non-randomized controlled trials [NRCTs] or cluster randomized trials [CRTs] were found on the topic, indicating a lack of research on the development and assessment of programs aimed at improving and maintaining sports persistence. However, this is significant for several reasons. Dropout prevention programs can assist individuals in establishing a consistent exercise routine, which can result in better cardiovascular health, improved muscular strength, enhanced coordination, and weight management. These programs also offer individuals the opportunity to cultivate and advance their athletic abilities. In terms of personality, persistence programs can encourage athletes to maintain a consistent effort and enable them to establish both short-term and long-term objectives. Sports programs have the potential to mold an individual’s character by instilling essential qualities such as discipline, perseverance, teamwork, and resilience. This can result in a long-term commitment to those activities. Additionally, participating in sports persistence programs can often cultivate a sense of camaraderie and community among its members, according to White et al.’s recent research.

### 4.1 Practical implications – how to prevent dropout

Preventing dropout from sports involves addressing various factors contributing to disengagement or dissatisfaction (Table 3). When focusing on dropout prevention, we should emphasize the athlete’s well-being and performance, motivation including competence, autonomy and relatedness, and resilience / mental toughness as well. At the same time, these may serve as preventing factors against dropout from sport, which belongs to the onto-system level. When focusing on individual factors, we should support the progress of adopting the personal pace while different players progress at different places throughout their sporting careers. This is natural, and do not force a peer comparison; even if the process has the same steps and structure, everyone goes their own way. Also, coping with anxiety is essential: “*We’re glad you are in the team, you are going through a tough time, but you are doing great work. I expect you to work hard in the coming weeks, but never overdo it*.” Regarding individual attention, goal setting is also crucial. What has changed since last week? What is the next step in your progress?

**Table 3:** The thoughts of the athletes related to some important scenes of the sporting career.

Damaged competence	Negative thought	Possible positive thought
Self-efficacy/Autonomy	“I do not influence how I perform or whether I will be in the starting line-up.”	“A difficult period is also part of a sporting career. Even in the ups and downs, I do everything professionally. The athlete who has come out of the slump is a mentally stronger team member. I can find the team, the level of the sport, where I can get a realistic challenge.”
Competence	“I am not able to do anything. I suck. I have not had a sense of achievement in a long time.”	
Identity	“If I’m not a successful athlete, who am I?”	
Relatedness	“I am excluded from the team. They do not like me here.”	“I know my limits, and I defend them. I know that I deserve respect, even if we do not build friendly relationships within the team.”
Mistrust	“Does the coach even know what I need?”	“I trust the process even if I do not see immediate progress.”
Strategy	“I train twice as much to be even better.”	“I will check with my coach to see what extra training will be effective for me.”

Concerning the micro-system levels, the valuable attention of the coach must be emphasized. The detection of negative and demotivating thoughts can be listed along with reframing, positive inner talk and thought stopping [stopping the negative cycle]. Also, building a positive and supportive climate [autonomy-supportive leadership style] is of paramount importance.

### 4.2 Limitations

Despite providing a thorough evaluation and contrast of the studies featured in the literature, it is important to acknowledge the limitations of this research. Specifically, the studies that were examined only focused on sport persistence, which may not provide a complete understanding of its growth and development. Furthermore, only cross-sectional studies were identified, which prevented us from investigating the dynamic nature of sport persistence. Our ability to identify programs that specifically target the development of sport persistence was also hindered by the lack of methodological diversity in the studies. Lastly, it is worth noting that our analysis was limited to English papers, which could have potentially impeded our ability to detect relevant programs.

## 5 Conclusion

Overall, when focusing on dropout prevention, some factors can be mentioned as variables predicting persistence, such as personality, positive emotions, enjoyment, satisfaction with sport and life and openness. Also, low levels of fatigue and health-related issues (e.g., injuries) and, parallelly, high levels of motivation and commitment, orientation, companionship, victory orientation, volition, future sporting intentions and learning and development may be predictive. Also, positive feedback, desire to learn and peer comparison can be listed.

Regarding the microenvironment, the coach can be supportive when using an autonomy-supportive leadership style and creating a task-involving motivational climate (greater competence, sense of autonomy). Concerning the peers, the positive presence of teammates should be emphasized. Parents and siblings are less and less important. Regarding the mezo and macro-environment, social expectations and gender differences should be pointed out. Intensive training, living with pain, muscle soreness and pushing performance limits may have a negative impact on persistence, and we can see lower drop-out rates in sports that can be played over the long term, even as a hobby. The development of emotional and motivational skills (emotion regulation, perception of abilities, development-oriented thinking, perseverance), optimism, an optimal level of competition, as well as geographical and material accessibility and social status can also be supportive.

Future studies should also focus on the topics detected as underrepresented themes to better understand the nature of dropout and persistence and our potentials to its development and maintenance. Furthermore, longitudinal investigations should be also carried out to reach a better understanding.

## Data Availability

The original contributions presented in the study are included in the article/supplementary material, further inquiries can be directed to the corresponding author.

## Footnotes

^1^ https://inplasy.com/inplasy-2024-11-0015/

^2^ https://doi.org/10.37766/inplasy2024.11.0015

## Appendix

**Table A1:** The evaluation of the risk of bias of the papers based on Joanna Briggs Institute (JBI) critical appraisal tool (49).

	Q1	Q2	Q3	Q4	Q5	Q6	Q7	Q8	Risk assessment
Baron-Thiene & Alferman (50)	Yes	Yes	Yes	Yes	Yes	Yes	Yes	Yes	Low
Calvo & Topa (51)	Yes	Yes	Yes	Yes	Yes	No	Yes	Yes	Moderate
Cervelló et al. (54)	Yes	Yes	Yes	Yes	Yes	Yes	Yes	Yes	Low
Consoni et al. (52)	Yes	Yes	Yes	Yes	Yes	Yes	Yes	Yes	Low
Duda (58)	Yes	Yes	Yes	Yes	No	No	Yes	Yes	Moderate
Guillet et al. (55)	Yes	Yes	Yes	Yes	Yes	Yes	Yes	Yes	Low
Fraser-Thomas et al. (60)	Yes	Yes	Yes	Yes	No	No	Yes	Yes	Moderate
Le Bars et al. (56)	Yes	Yes	Yes	Yes	Yes	Yes	Yes	Yes	Low
Lea & Branco (53)	No	No	Yes	Yes	Yes	Yes	Yes	Yes	Low
Ryan et al. (59)	No	No	Yes	Yes	No	No	Yes	Yes	Moderate
Sarrazin et al. (57)	Yes	Yes	Yes	Yes	Yes	Yes	Yes	Yes	Low

Questions of JBI are as follows: Q1. Were the criteria for inclusion in the sample clearly defined? Q2. Were the study subjects and the setting described in detail? Q3. Was the exposure measured in a valid and reliable way? Q4. Were objective, standard criteria used for measurement of the condition? Q5. Were confounding factors identified? Q6. Were strategies to deal with confounding factors stated? Q7. Were the outcomes measured in a valid and reliable way? Q8. Was appropriate statistical analysis used?
